# Das Nationale Gesundheitsportal: Entwicklungs- und Nutzungspotenziale unter besonderer Berücksichtigung der hausärztlichen Perspektive

**DOI:** 10.1007/s00103-021-03288-y

**Published:** 2021-02-13

**Authors:** Julian Wangler, Michael Jansky

**Affiliations:** Zentrum für Allgemeinmedizin und Geriatrie, Universitätsmedizin Mainz, Am Pulverturm 13, 55131 Mainz, Deutschland

**Keywords:** Gesundheitsportal, Gesundheitsinformation, E‑Health, Gesundheitskommunikation, Gesundheitskompetenz, Arzt-Patienten-Verhältnis, Health portal, Health information, e‑Health, Health communication, Health literacy, Doctor-patient relationship

## Abstract

**Hintergrund:**

Das in Deutschland eingerichtete Nationale Gesundheitsportal soll evidenzbasierte Gesundheitsinformationen bereitstellen. Es erscheint sinnvoll, Entwicklungsperspektiven zu reflektieren, um Anwendungshorizonte, Nutzungs- und Wirkungspotenziale des Portals abzuschätzen und Mehrwerte generieren zu können.

**Ziel der Arbeit:**

Unter Berücksichtigung von Implementierungserfahrungen anderer europäischer Länder sollen der Sachstand des Vorhabens sowie die Perspektive von politischen Entscheidungsträger*innen und Hausärzt*innen umrissen werden. Aus der Analyse sollen Empfehlungen abgeleitet werden, welche Ausgestaltung für ein nationales Gesundheitsportal insbesondere mit Blick auf die hausärztliche Versorgung Erfolg versprechend erscheint.

**Methoden:**

Anhand einer Literaturrecherche sowie auf Basis zweier Vorstudien geht der Artikel der Frage nach, in welchen Entwicklungskategorien ein nationales Gesundheitsportal perspektivisch ausgestaltet werden könnte.

**Ergebnisse:**

Auf Basis der Zusammenschau erscheint eine Reihe von Dimensionen ausschlaggebend, entlang derer sich Grundsatzentscheidungen zur Ausrichtung des Nationalen Gesundheitsportals treffen lassen. Diese beziehen sich auf die Einbettung in eine E‑Health-Strategie, Konvergenz- und Integrationsmaßnahmen hinsichtlich evidenzbasierter Informationen, die Sicherstellung von Auffindbarkeit, die inhaltliche Schwerpunktsetzung und Zielgruppenansprache, die funktionelle Ausgestaltung sowie die Trägerschaft und Einbeziehung von Gesundheitsakteuren.

**Diskussion:**

Ein evidenzbasiertes Portal kann ein wertvolles Instrument sein, um Patient*innen besser aufzuklären, das Arzt-Patienten-Verhältnis zu stärken und die Digitalisierung des Gesundheitswesens voranzutreiben. Bereits während der Initiierungsphase sollten ein Innovations- und Integrationspotenzial sowie eine ausreichende Bekanntheit und Sichtbarkeit innerhalb des Gesundheitswesens sichergestellt werden.

## Abstract (en)

**Background:**

The German National Health Portal, which is currently in preparation, is intended to provide evidence-based health information. It seems sensible to reflect on development perspectives in order to be able to estimate application horizons as well as usage and impact potential of the portal under the conditions of the health system so that specific additional value can be generated.

**Objectives:**

Considering the implementation experience of other European countries, the current status of the project and the perspective of political decisionmakers and medical practitioners are outlined. Fundamental recommendations on how a national health portal can be successfully established, especially with regard to primary care, are derived from the analysis.

**Methods:**

On the basis of a literature search and two preliminary studies, the article examines the development categories in which a national health portal could be designed in the future.

**Results:**

Based on the overview, a number of dimensions appear to be decisive for the planning and implementation process of a national health portal. These relate to embedment in a higher-level e‑health strategy, convergence and integration measures with regard to evidence-based information, ensuring sufficient findability on the Internet, a substantive focus that addresses target groups, a functional structure, as well as sponsorship and involvement of healthcare actors.

**Conclusions:**

An evidence-based portal can be a valuable instrument to better educate patients, strengthen the doctor–patient relationship, and advance the digitalization of the healthcare system more strongly. Therefore, a sufficient degree of innovation and integration ability as well as awareness and visibility within the health system should be ensured.

## Einleitung

Angesichts einer großen Zahl von Menschen, die sich im Internet über Krankheitsbilder, Diagnosen und Therapien informieren, kommt digitaler Gesundheitskompetenz große Bedeutung zu, also der Fähigkeit, relevante Gesundheitsinformationen über E‑Health-Angebote zu recherchieren, zu beurteilen und zur Förderung der eigenen Lebensqualität anzuwenden [1]. Um informierte Gesundheitsentscheidungen zu fördern, sind evidenzbasierte, qualitätsgesicherte Informationsangebote unverzichtbar [2].

Trotz eines enormen Angebots digitaler Gesundheitsinformationen zeigen Studien, dass viele Plattformen nicht den Anforderungen an vertrauenswürdige Patienteninformationen entsprechen [3, 4]. Hierzu zählen u. a. wissenschaftliche Fundiertheit und Aktualität, aber auch Neutralität, Ausgewogenheit und allgemeine Verständlichkeit von Informationen, daneben die Diskussion von Vor- und Nachteilen diagnostischer und therapeutischer Optionen sowie transparente Quellenverweise [5]. Infolgedessen sind viele digitale Angebote zur Gesundheitsinformation der Vorbereitung und Herbeiführung informierter Entscheidungen nicht dienlich [1].

Eine zweite Problematik besteht in der mangelnden Auffindbarkeit qualitätsgesicherter Gesundheitsinformationen, wodurch sie Patient*innen, aber auch Ärzt*innen oftmals nicht geläufig sind [6]. In den zurückliegenden Jahren hat sich gezeigt, dass es nichtkommerziellen, evidenzbasierten Gesundheitsportalen nicht gelungen ist, einen hohen Grad an Bekanntheit zu erlangen [7]. Dies hängt damit zusammen, dass Patient*innen verbreitet über Suchmaschinenrecherchen agieren und in der Trefferliste werbefinanzierte Anbieter erheblich prominenter vertreten sind [8]. Die Recherche mittels Suchdienst birgt für unerfahrene Nutzer die Gefahr, auf unseriöse Seiten zu geraten und durch inkorrekte Informationen verunsichert zu werden [9]. Studien zufolge hat mehr als die Hälfte der Bevölkerung Schwierigkeiten, sich gesundheitsrelevante Informationen zu beschaffen und zu bewerten [10].

Um die angesprochenen Problemkreise zu adressieren, wurde unter Federführung des Bundesministeriums für Gesundheit (BMG) die Einrichtung einer nationalen Gesundheitsplattform festgeschrieben [11, 12]. Im Vorfeld hatten sich u. a. der Sachverständigenrat Gesundheit und die Allianz für Gesundheitskompetenz für ein leicht auffindbares, qualitätsgeprüftes Portal ausgesprochen, das frei zugängliche, verständliche und neutrale Gesundheitsinformationen zur Verfügung stellt [2].

Die für den Regelbetrieb ab 2021 vorgesehene Gesundheitsplattform, die seit November 2020 im Probebetrieb unter www.gesund.bund.de zugänglich ist, folgt dem Beispiel einzelner europäischer Länder [13, 14]. Trotz Initiierung der Umsetzungsphase ist für das deutsche Portal noch teilweise offen, in welcher Weise es einen Beitrag zur Gesundheitskompetenz leisten und worauf sein Schwerpunkt liegen soll. Es erscheint wichtig, Zielstellungen, Kriterien und Fokusse zu definieren, damit das neue Angebot Alleinstellungsmerkmale besitzt und Bedürfnissen im Gesundheitswesen entgegenkommt.

## Methodik

Der Überblicksartikel geht anhand einer Literaturrecherche sowie zweier Vorstudien [15, 16] der Frage nach, in welchen Entwicklungskategorien ein nationales Gesundheitsportal in Deutschland perspektivisch ausgestaltet werden könnte, um Mehrwerte bereitzustellen. Unter Berücksichtigung ausgewählter nationaler Portale in anderen europäischen Ländern sollen Informationen zusammengetragen werden, die den Sachstand des Vorhabens, die Perspektive politischer Entscheider und (primär-)ärztlicher Versorger mit Blick auf das zu schaffende Angebot herausarbeiten.

Hierbei liegt ein Schwerpunkt auf der hausärztlichen Perspektive, da die erwähnten Vorstudien bis dato die einzigen Arbeiten im deutschsprachigen Raum darstellen, die Einstellungen einer bestimmten Akteursgruppe im Gesundheitssystem zum genuinen Konzept eines nationalen Gesundheitsportals einholen. Bei den Studien handelt es sich um eine Fokusgruppe mit 11 Hausärzt*innen sowie um eine Befragung von 745 Hausärzt*innen (Rheinland-Pfalz, Saarland). Die Literaturrecherche beruht auf einer PubMed- sowie Google-Suche, bei denen der Begriff „(national) (e-)health portal“ einzeln oder in Kombination mit anderen Schlüsselwörtern dieses Beitrags vorkam. Die Perspektive politischer Entscheider wurde v. a. durch eine Recherche in den Debattenprotokollen des Deutschen Bundestages sowie Meldungen des BMG abgedeckt. Auch Medienreportagen wurden hinzugezogen. Als Vergleichsländer wurden westeuropäische Länder ausgewählt, die bei der Entwicklung eines nationalen Gesundheitsportals vergleichsweise fortgeschritten sind.

Aus der Analyse sollen grundlegende Empfehlungen abgeleitet werden, welche Ausgestaltung für ein nationales Gesundheitsportal in Deutschland aussichts- und chancenreich erscheint. Zugleich sollen Möglichkeiten, aber auch Grenzen des Konzepts erkennbar werden.

## Ergebnisse

**Figure 1:**
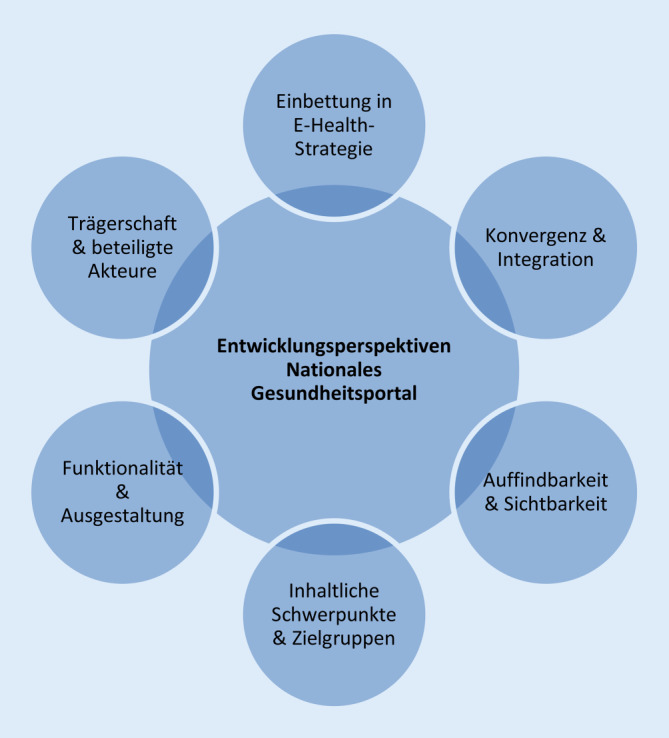

Abb. 1 zeigt die im Zuge der Recherche herausgearbeiteten Dimensionen, die für die Entwicklungsperspektive eines nationalen Gesundheitsportals relevant erscheinen und distinkte Pfade der Konzeption eröffnen. Je nach Ausprägung dieser Parameter kann das Portal unterschiedliche Rollen und Funktionen wahrnehmen. Im Folgenden sollen besagte Dimensionen näher beleuchtet werden.

### Einbettung in E-Health-Strategie

Sowohl im Koalitionsvertrag [11] als auch in der Digitalstrategie der Bundesregierung [17] findet das Nationale Gesundheitsportal Erwähnung. Ausgehend von 5 Handlungsfeldern soll es v. a. für eine bessere Aufklärung der Bevölkerung bei Gesundheits- und Krankheitsthemen sorgen. Allerdings ist zum jetzigen Zeitpunkt noch nicht klar zu erkennen, welchen Platz die zu schaffende Plattform im Rahmen der Digitalstrategie einnehmen soll oder ob sie längerfristig stärker als für sich stehendes Einzelangebot fungieren wird.

Schaut man nach Dänemark, so zeigt sich, dass das seit 2003 systematisch etablierte Portal *sundhed.dk* frühzeitig als Bestandteil einer nationalen E‑Health-Strategie definiert wurde. Letztere ist bereits in den 1990er-Jahren in einem Konsens aller zentralen Akteure im dänischen Gesundheitssystem verabschiedet worden [18]. Im Zuge mehrerer Ausbaustufen wurden die Zielstellungen mit Blick auf *sundhed.dk* sukzessive angepasst. Das Portal steht heute im Zentrum der E‑Health-Strategie, da es verschiedene Bestandteile miteinander verbindet (E-Rezepte, elektronische Patientenakte, Arzt-Patient-Kommunikation, Leistungen des Gesundheitsdienstes, Organspende etc.; [13, 18]). Dies hatte zur Folge, dass *sundhed.dk *sich immer mehr zu einer komplexen Leistungserbringungsplattform gewandelt hat. Demgegenüber ist das im Vereinigten Königreich eingesetzte Gesundheitsinformationsportal *NHS Choices* (NHS: National Health Service) ein Beispiel für eine Lösung, bei der ein nationales Portal nicht in eine kohärente E‑Health-Strategie eingebettet ist, was mit dem Fehlen selbiger auf Regierungsseite zusammenhängt [14]. Nach eigener Aussage zielt *NHS Choices* in erster Linie darauf ab, die Gesundheitskompetenz von Patient*innen durch ein breites Informationsangebot zur Funktionsweise des NHS und zu dessen Dienstleistungen, aber auch zu Krankheiten und Behandlungen zu erhöhen [19].

Die Stellungnahmen der deutschen Bundesregierung lassen vermuten, dass das Nationale Gesundheitsportal weniger als vorrangiges Element der Digitalstrategie wahrgenommen wird als vielmehr zu deren Ermöglichung und Flankierung. So wird das Portal im Rahmen einer Kleinen Anfrage im Bundestag als eine von mehreren wichtigen „Voraussetzungen“ beschrieben, damit die Bevölkerung mithilfe digitaler Angebote „relevante Gesundheitsinformationen finden und … verstehen“ und somit einschätzen könne, „ob diese Informationen für die eigene gesundheitliche Entscheidungsfindung nutzen können“ [20].

### Konvergenz und Integration

Eine wichtige Frage ist, ob eine der primären Eigenschaften eines nationalen Gesundheitsportals darin bestehen soll, die inzwischen stark fragmentierte Vielfalt existierender Informationsangebote im Internet stärker zu integrieren oder ob sich das Portal auf eigenen Inhalt konzentriert [15]. Im ursprünglichen Konzept des Instituts für Qualität und Wirtschaftlichkeit im Gesundheitswesen (IQWiG) war zunächst vorgesehen, dass das zu schaffende Portal „vor allem auf die Zusammenführung von bereits vorhandenem Inhalt qualitätsgeprüfter externer ‚Contentpartner‘ setzt“ [12, 21]. In diesem Sinne sollte eine wesentliche Leistung des Portals sein, unter Zuhilfenahme einer kompetenten Redaktion und medizinischer Experten Seriositätsfaktoren zu prüfen und auf dieser Grundlage ein Netzwerk von assoziierten Seiten aufzubauen, die Synergien entfalten können. Verschiedene Verbände und Fachgesellschaften fordern ebenfalls, dass das Portal „die Pluralität *aller *vertrauenswürdigen evidenzbasierten Informationen garantiert“ [22]. Mit *NetDoktor.de* und *Apotheken-Umschau.de* hatten die beiden potenziell größten Konkurrenzseiten frühzeitig eine Kooperation mit dem Nationalen Gesundheitsportal ins Gespräch gebracht [21]. Laut BMG soll jedoch ein vollständig eigenständiges Informationsvollangebot etabliert werden, um „Fragen der Nutzer zu beantworten und ihnen Wege durch den Dschungel der Gesundheitsversorgung in Deutschland zu weisen“ [21].

Betrachtet man die in anderen europäischen Ländern bereits eingerichteten Portale, zeigt sich, dass in sämtlichen Fällen der Anspruch verfolgt wurde, ein Portal mit eigengenerierten Inhalten aufzuziehen und nicht die Leistungen anderer evidenzbasierter Anbieter zu fusionieren [13, 14]. Eine solche Festlegung erfordert einen deutlich größeren Ressourcenaufwand und bringt die Herausforderung mit sich, dass es zu einem direkten Wettbewerb mit etablierten Anbietern kommt. Langfristig eröffnet das Konzept eines inhaltlich autarken Informationsportals allerdings auch die Möglichkeit, funktionelle Erweiterungen vorzunehmen, die den Abruf verschiedener Gesundheitsdienstleistungen betreffen. In diesem Zusammenhang behält es sich auch das BMG explizit vor, an die Schaffung eines Gesundheitsinformationsangebots neue Entwicklungsetappen anzuschließen, die beispielsweise in Richtung einer Behandlungsmanagement- und Beratungsplattform gehen können [12, 21].

### Auffindbarkeit und Sichtbarkeit

Der Erfolg des Nationalen Gesundheitsportals wird maßgeblich davon abhängen, inwiefern es leicht auffindbar und sichtbar sein wird. Daher wird ausschlaggebend sein, auf welchem Weg Nutzer*innen eine solche Plattform erreichen. Bislang geschieht dies verbreitet über die Schlagwortrecherche via Suchmaschine [9]. Zwar dürfte ein Portal des Bundes nach den Kriterien, die Suchmaschinen derzeit anlegen, ein besseres Ranking erzielen als andere öffentlich-evidenzbasierte Gesundheitsseiten, allerdings wird dieser Vorteil allein möglicherweise nicht ausreichen. Insofern wäre eine Möglichkeit, stärkere Investitionen in das Suchmaschinenranking vorzunehmen und die Kooperation mit Suchdiensten auszubauen, um in der Suchhistorie möglichst prominent berücksichtigt zu sein. Eine andere Option wäre, durch gezielte Kommunikationsmaßnahmen nachhaltig auf das neue Angebot aufmerksam zu machen und systematisch Nutzervertrauen aufzubauen, sodass das Portal bei der Bevölkerung als Anlaufstelle verankert ist.

In Dänemark ist es mit *sundhed.dk* gelungen, einen hohen Grad an Bekanntheit, Zuspruch und Nutzerbindung zu erzielen. Nach dem Relaunch auf einer neuen technischen Plattform 2009 nahmen die Besucherzahlen des Portals nochmals 50 % zu, sodass inzwischen rund 1,7 Mio. Menschen jeden Monat *sundhed.dk *nutzen [23]. Hinzu kommt, dass sämtliche Dän*innen mit der Geburt eine persönliche Identifikationsnummer zugewiesen bekommen, über die zeitlebens ein Abruf der kompletten Krankengeschichte möglich ist. Im Vereinigten Königreich ist es wiederum gelungen, mit *NHS Choices* Bekanntheit und Ansehen des NHS zu transponieren, sodass das Portal weiten Bevölkerungskreisen ohne Zuhilfenahme einer Suchmaschine bekannt ist [14, 19].

Bei der Vorstellung von www.gesund.bund.de im November 2020 hat sich gezeigt, dass über eine Zusammenarbeit mit Google die Einrichtung eines sogenannten Knowledge-Panels vereinbart wurde, das als separates Fenster kompakte Informationen des Gesundheitsportals prominent präsentiert [24]. Zudem sind weitergehende Schritte geplant, um innerhalb des Suchmaschinenrankings Präsenz zu gewinnen und das Portal als „Alternative zur einfachen Suche mittels Suchmaschine“ aufzubauen [7, 8].

### Inhaltliche Schwerpunkte und Zielgruppen

Ein basaler Fragekomplex ist, welche inhaltlichen Schwerpunkte das nationale Portal setzen und welche Bevölkerungszielgruppen es ansprechen soll. Damit verbunden stellt sich die Frage, inwieweit sich die Plattform streng am Behandlungspfad orientiert und Patient*innen den Strukturen des Gesundheitssystems zuweist. Entsprechend ist im Hinblick auf die Ausrichtung die Grundsatzentscheidung zu treffen, ob das Portal ein „Hilfsmittel für den eigenverantwortlichen Alltag“ oder ein „mehr oder weniger dezidierter Lotse durch das Gesundheitssystem“ sein soll [15].

Analog zur Einbettung in nationale Gesundheits- und Digitalstrategien sind die in anderen europäischen Staaten entstandenen Portale primär ein Utensil zum effektiveren Patient*innen-Management. Nicht nur das dänische *sundhed.dk*, sondern auch seine norwegischen und schwedischen Pendants *HealthNorway *und *1177.se *betonen für die gesamte Bevölkerung den niedrigschwelligen Abruf spezifischer Dienste, wie z. B. elektronische Fallakten oder die digitale Unterstützung der Arzt-Patient-Kommunikation [13, 25].

Für das in Deutschland einzurichtende Portal scheint festzustehen, dass eine „breite Zielgruppenausrichtung“ gewählt werden und eine bevorzugte Ansprache bestimmter Nutzerkreise eher vermieden werden soll [20, 21]. Hingegen ist noch nicht abzusehen, wie stark ein solches Portal versuchen soll, Patient*innen gezielt und systematisch dem Versorgungswesen zuzuführen, oder demgegenüber ihre Autonomie betont, also ggf. auch dabei behilflich sein soll, Tipps und Lösungen abseits des üblichen Behandlungswegs anzubieten.

Die in diese Analyse einbezogene Befragung von 745 Hausärzt*innen in Südwestdeutschland zeigt, dass Hausärzt*innen sich einen offenen Ansatz für das Portal wünschen [16]. Vor allem die Dimension der Prävention und Gesundheitsförderung solle in Kombination mit einer symptomorientierten Darstellung betont werden. Dabei hält es ein beträchtlicher Teil der Hausärzt*innen für wünschenswert, wenn Patient*innen auf dem Portal im Umgang mit dem Gesundheitswesen „geschult“ werden, um Versorgungsleistungen möglichst angemessen zu nutzen. Auch erhoffen sich Hausärzt*innen, dass ein nationales Gesundheitsportal zur psychosozialen Stabilisierung von gesundheitsängstlichen Patient*innen beiträgt. In einer Fokusgruppe haben die einbezogenen Ärzt*innen angeregt, dass das Portal passgenau auf die Recherchemotive von Patient*innen eingehen sollte, die nicht unbedingt Information, sondern auch Bestätigung und Beruhigung suchen [15].

### Funktionalität und Ausgestaltung

Angesichts einer Vielzahl existierender Gesundheitsinformationsseiten wäre zu bedenken, inwiefern auf einem nationalen Portal von Eigenschaften wie Individualisierbarkeit und Interaktivität Gebrauch gemacht werden könnte. Hierzu zählen visuelle Formen der Informationsvermittlung, individualisierbare Recherchemöglichkeiten, die Anbindung an soziale Netzwerke, Angebote der künstlichen Intelligenz, die Einbeziehung von Gesundheits-Apps und (algorithmischen) Diagnosetools, die bei der Vermittlung zu einzelnen Strukturen im Gesundheitssystem behilflich sein könnten. Wichtig erscheint, dass die Funktionslogik eines solchen Portals „von Anfang an hinreichend ‚anpassungsoffen‘ ist“, um sich auf verändernde Interessen und Erfordernisse einzustellen [26].

In anderen europäischen Ländern ist die Adaptivität nationaler Gesundheitsportale zumeist im Vorfeld mitbedacht worden, um Entwicklungshorizonte auszuloten [14]. Dies gilt nicht nur für den schrittweisen Ausbau des dänischen Beispiels zu einer Versorgungsplattform. Auch in Schweden ist ein Ansatz verfolgt worden, der über die Bereitstellung von Informationen und die Inanspruchnahme digitaler Gesundheitsdienstleistungen (u. a. Terminbuchungen, Testergebnisse, Videokonsultationen, E‑Patientenakte, Beratungsangebote) hinausgeht und z. B. auf Konnektivität und Interaktion mit lizensierten Gesundheits-Apps setzt [13]. Damit in Zusammenhang stehen vielfältige Möglichkeiten des Austausches und Monitorings zwischen Patient*innen und Ärzt*innen sowie anderen Gesundheitsakteuren. Ähnliches gilt für die norwegische E‑Health-Plattform *HealthNorway*, die den digitalen Dialog ebenfalls betont [25].

Vor dem Hintergrund solcher Beispiele plant das BMG, mit dem deutschen Portal „eine wichtige Referenzfunktion für die Kommunikation zwischen Ärzt*innen und Patient*innen“ zu besetzen [20]. Hierbei sollen Patient*innen mithilfe von verständlichen und eingängigen Grafiken und Erklärvideos angesprochen und die Wissensvermittlung unterstützt werden. Funktionell solle sich das Portal in erster Linie an Laien richten, allerdings mit möglicherweise erweiterten und speziellen Funktionen durchaus auch von medizinischen Profis zur gezielten Aufklärung von Patient*innen genutzt werden [16, 20].

Die Befragung niedergelassener Allgemeinärzt*innen hat gezeigt, dass diese sich ein seriöses, symptomorientiertes Informationsportal wünschen, das Patient*innen Gesundheitswissen über zeitgemäße und visuelle Präsentationsformen einprägsam vermittelt und so die Arzt-Patient-Kommunikation stärkt. Für einen Teil der Hausärzt*innen ist vorstellbar, über eine solche Plattform in einer separaten Sektion zwecks zielgerichteter Beratung individualisierbare Informationen für Patient*innen zusammenzustellen (45 %). Ein ähnlich hoher Anteil (44 %) würde gerne den Austausch mit Fachkollegen über das Portal suchen [16].

### Trägerschaft und beteiligte Akteure

Schließlich stellt sich für die Ausrichtung des Nationalen Gesundheitsportals die Frage, welche Akteure an seiner (Weiter‑)Entwicklung und Administration beteiligt werden, um Expertise einzuspeisen sowie Evidenzbasierung sicherzustellen. Relevant ist auch, ob eine zentrale Steuerung seitens eines einzigen Trägers erfolgt oder ob das Portal dezentral ausgerichtet wird. Ferner ist der Gesichtspunkt der organisatorischen Trennung von Konzeption und Erstellung der Inhalte anzusprechen.

Eine der Leistungen der dänischen Gesundheitspolitik bestand darin, zahlreiche Stakeholder des Gesundheitssystems unter einer Digital-Health-Strategie zu versammeln, die für *sundhed.dk* spezifiziert wurde. Dadurch wurden eine wirksame Qualitätskontrolle und eine Vielfalt des Angebots realisiert [23]. Zugleich wurde mittels Einbindung von Regionen, Kommunen und Unternehmen sowie mit dem Betrieb durch eine Non-Profit-Organisation der Eindruck eines politisch gesteuerten Portals vermieden [18].

Das BMG hat sich dazu entschieden, das Portal vorerst im eigenen Haus zu verankern und alleiniger Träger zu sein [20]. Dazu haben sich mehrere Fachgesellschaften und Verbände kritisch geäußert. So moniert das Deutsche Netzwerk Evidenzbasierte Medizin, es sei „nicht ersichtlich, wie das BMG die Expertise aufbauen könnte, umfassende Gesundheitsinformationen bereitzustellen, die auf bester Evidenz fußen“ [27]. Vielmehr bestehe nun die Gefahr, dass das BMG als Träger „politische Steuerung statt informierter Entscheidungen“ bewirke [27].

Jenseits der Trägerschaft ist für den Erfolg eines Portals hoch relevant, welche Gruppen im Versorgungssystem gezielt angesprochen und in der Rolle von Multiplikatoren für ein nationales Gesundheitsportal einbezogen werden können. Das BMG hat angekündigt, als Partner für das Portal zunächst mit dem IQWiG, dem Deutschen Krebsforschungszentrum und dem Robert Koch-Institut zusammenarbeiten zu wollen [21, 24].

Als Assoziierte eines solchen Portals wären auch niedergelassene Ärzt*innen denkbar. Die Hausärztebefragung hat gezeigt, dass Allgemeinmediziner*innen unter der Voraussetzung eines seriösen, evidenzbasierten Portals Gebrauch von Verweisen und Empfehlungen im Patientengespräch machen würden [15, 16]. Im Gegenzug besteht der Wunsch, dass das Portal ausreichend Konformität mit der Primärversorgung bietet, weshalb eine Einbindung in den Entwicklungsprozess für sinnvoll erachtet wird [16].

## Diskussion

Bislang gibt es in Deutschland für viele gesundheitsbezogene Themen keine leicht verständlichen, evidenzbasierten Informationsmaterialien; Ähnliches gilt für übersichtliche Entscheidungshilfen [1, 2]. Ein nationales Gesundheitsportal kann dem Gesundheitswesen neue Möglichkeiten eröffnen, die sich nicht nur auf evidenzbasierte Aufklärung der Bevölkerung zu Gesundheits- und Krankheitsthemen beziehen, sondern im Idealfall auch Vorteile im Versorgungsprozess mit sich bringen können. Diese Mehrwerte können ebenso Fragen der partizipativen Entscheidungsfindung betreffen als auch Compliance-Effekte, eine raschere und konsequentere Betreuung, Diagnostik bzw. Behandlung sowie ein effektives Abrufen digitaler Gesundheitsdienstleistungen [2, 11, 12]. Versorger wie Hausärzt*innen können angesichts des hohen Vertrauens, das sie bei ihren oft langjährigen Patient*innen genießen, bei der Kommunizierung und gezielten Anwendung seriöser Gesundheitsseiten eine wichtige Multiplikator- und Begleitfunktion ausüben und so zu einer besseren Information und Beratung von Patient*innen beitragen [9]. Dennoch muss auch auf mögliche Risiken und Überforderungssituationen von Verbraucher*innen hingewiesen werden, denn je komplexer die Funktionalität eines nationalen Gesundheitsportals ausgestaltet wird, desto größer werden Kompetenzanforderungen an Nutzer*innen sowie Anforderungen an die Sicherheitsarchitektur (z. B. Umgang mit personenbezogenen Daten; [10]).

Der Schwerpunkt der Übersichtsarbeit liegt auf der hausärztlichen Perspektive, konkret auf 2 Studien mit (regional) begrenztem Sample. Obwohl auch Sichtweisen anderer Akteure berücksichtigt werden, waren die Autoren mit der Herausforderung konfrontiert, dass jenseits der erwähnten Hausärztebefragungen keine empirischen Arbeiten vorgelegt wurden, die Positionen oder Erfahrungen von Gesundheitsstakeholdern zum Thema beleuchten. Vor diesem Hintergrund ist zu fragen, zu welchem Grad die Einstellungen von Hausärzt*innen auf Beschäftigte des Gesundheitswesens übertragen werden können. Zudem können die Ergebnisse nicht ohne Weiteres auf die Bedürfnisse und Interessen der Bevölkerung verallgemeinert werden. Entsprechend ist kritisch zu fragen, inwiefern über ein nationales Portal denkbare Features (z. B. Kommunikation mit Ärzt*innen, Diagnosetools) den Bevölkerungsinteressen entgegenkommen. Konkrete Erkenntnisse hierzu stehen noch aus.

Allerdings zeigt eine gesamteuropäische Studie, dass die Bevölkerung in Deutschland im Vergleich mit Leistungserbringern aufgeschlossener gegenüber digitalen Lösungen ist [28]. So versprechen sich mehr als 3 von 4 Bürger*innen große Vorteile von der Anwendung von Vorsorge- und Monitoring-Apps. Auch werden der Wunsch und Bedarf nach einer besseren Strukturierung und Orientierung digitaler Hilfsmittel im Gesundheitswesen zum Ausdruck gebracht. Die Studie macht als Ansatz zur Adressierung dieses Bedarfs eine digitale Plattform aus, um Akteure, Informationen und digitale Gesundheitsservices nach festgelegten Sicherheitsstandards zu vernetzen.

### Schlussfolgerungen

Auf Grundlage der Zusammenschau erscheint eine Reihe von Dimensionen ausschlaggebend, entlang derer sich der Planungs- und Umsetzungsprozess für ein nationales Gesundheitsportal beschreiten und Grundsatzentscheidungen zur Ausrichtung treffen lassen. Aus der Analyse, die primär die hausärztliche Perspektive berücksichtigt, wurden Ansatzpunkte abgeleitet, die bei der Konzeption erwogen werden sollten, um positive Effekte im Gesundheitswesen zu erzielen:

#### Einbettung in E-Health-Strategie.

Es erscheint überlegenswert, das Portal in eine längerfristig angelegte, übergreifende Digitalstrategie einzufügen. Im Rahmen einer ausformulierten E‑Health-Strategie könnte das Portal integrativ wirken, indem verschiedene Leistungen gebündelt werden. Die Verbindung zwischen einrichtungsübergreifenden elektronischen Patientenakten und der Vermittlung qualitätsgeprüfter, verständlicher Informationen könnte zu einer effektiven Behandlungsmanagementplattform mit hoher Nutzerbindung führen.

#### Konvergenz und Integration.

Das Vorhaben, mit dem Nationalen Gesundheitsportal ein eigenständiges redaktionelles Angebot schaffen zu wollen, erscheint nachvollziehbar. Allerdings sollte auch darüber nachgedacht werden, Synergieeffekte mit bereits bestehenden (öffentlich-evidenzbasierten) Portalen zu nutzen, indem etwa die Möglichkeit einer Kooperation und Vernetzung ausgelotet wird. Auf diese Weise kann die Präsenz qualitativ hochwertiger Gesundheitsinformationen effizient gestärkt werden [21].

#### Auffindbarkeit und Sichtbarkeit.

Ohne ein ausreichendes Maß an öffentlicher Sichtbarkeit wird das Portal seinem Anspruch als „zentrales Informationsangebot für Gesundheitsfragen“ kaum gerecht werden können [12]. Daher werden Maßnahmen zur Bekanntheitssteigerung und Vertrauensgenerierung in der Bevölkerung unverzichtbar sein. Eine wichtige Rolle können dabei (niedergelassene) Ärzt*innen spielen [9].

#### Inhaltliche Schwerpunkte und Zielgruppen.

Insgesamt besteht die Herausforderung, dass bereits umfassende Angebote zur Gesundheitsinformation existieren. Daher stellt sich mit Blick auf das inhaltliche Profil eines nationalen Gesundheitsportals die Frage nach Alleinstellungsmerkmalen. Denkbar wäre, dass die Inhalte streng entlang des Behandlungspfads ausgerichtet sind und damit zu konkreten Strukturen und Angeboten des Versorgungssystems weiterleiten. Zudem könnte ein Portal abseits der Strukturen des Gesundheitswesens Hilfestellung für den Alltag vermitteln. Ebenso relevant ist es, mit dem Portal die richtige Ansprache zu finden und die Recherchemotive von Patient*innen (z. B. Bestätigung, Beruhigung, Trost) zu antizipieren [15, 22]. Qualitätsstandards zur Erreichung eines hohen Maßes an subjektiver Zufriedenheit bei der Onlinegesundheitsrecherche sind bereits erarbeitet worden [29].

#### Funktionalität und Ausgestaltung.

Um zu verhindern, dass ein traditionell konzipiertes Gesundheitsportal an den Verbraucherbedürfnissen vorbeigeht, sollten Elemente der Interaktivität und Individualisierbarkeit einbezogen werden. Befragungen von Patient*innen zeigen, dass diagnostische Tools, die bei der (Vorab‑)Einordnung von Symptomatiken behilflich sind und kontextsensitiv (u. a. Alter, Geschlecht, Vorerkrankungen) zu Informationen weiterleiten, positiv gesehen werden [13, 28]. Darüber hinaus könnte ein nationales Angebot zur Gesundheitsinformation technologische Entwicklungen aufgreifen und befördern. Hier wäre längerfristig denkbar, dass das Portal einen Überblick über seriöse Gesundheits-Apps gibt, in einem gesonderten, entsprechend geschützten Bereich das Hochladen gemessener Daten und deren Weiterleitung an Ärzt*innen sowie den Austausch mit selbigen ermöglicht.

#### Trägerschaft und beteiligte Akteure.

Für die Akzeptanz in der Bevölkerung wird es darauf ankommen, dass das Portal nicht als Instrument der Gesundheitspolitik wahrgenommen wird. Falls das BMG dauerhaft als Träger fungieren will, erscheint die Einrichtung eines unabhängigen Beirats geboten, der bei der Ausgestaltung des Portals Mitspracherecht hat [23]. Zudem wäre anzuregen, ein Netzwerk an Stakeholdern an das Portal zu binden, damit hier nicht nur Erfahrungen, Bedürfnisse und Rückmeldungen besser einfließen können, sondern auch die Wahrscheinlichkeit vergrößert wird, dass Informationen, z. B. in der Beratung von Patient*innen, aufgegriffen und genutzt werden. Hier sollte auch und gerade an Intermediäre wie Hilfs- und Beratungseinrichtungen gedacht werden. Auch der Einbezug von Fachgesellschaften erscheint sinnvoll.

Ungeachtet der Potenziale, die ein nationales Gesundheitsportal bietet, liegt ein Risiko in der Überfrachtung mit Ansprüchen an eine möglichst vielfältige Funktionalität, was zu einer Verkomplizierung der Nutzung, steigenden Kosten und zeitlichen Verzögerungen führen kann. Insofern sollten funktionale Zielsetzungen und Prioritäten möglichst klar und sukzessive umrissen werden. Auch kann ein solches Portal lediglich ein Baustein sein, um die Gesundheitskompetenz von Bürger*innen zu fördern, und wird nicht alle Bevölkerungsgruppen gleichermaßen erreichen können. Ebenso wenig ist es in der Lage, eingespielte Mechanismen der Informationssuche im Internet oder bestehende Marktlogiken zu durchbrechen [1, 6].

## Fazit

Das Nationale Gesundheitsportal soll Bürger*innen neutrale, evidenzbasierte Informationen zu Gesundheits- und Krankheitsthemen sowie zum Gesundheitssystem in Deutschland zur Verfügung stellen. Es kann dem Gesundheitswesen neue Möglichkeiten eröffnen, die sich nicht nur auf Information und Aufklärung der Bevölkerung zu Gesundheits- und Krankheitsthemen beziehen, sondern auch Vorteile im Versorgungsprozess mit sich bringen können (z. B. partizipative Entscheidungsfindung, Compliance-Effekte, konsequentere Diagnostik bzw. Behandlung).

Im Zuge der Entwicklung eines nationalen Gesundheitsportals sollte ein ausreichendes Innovations- und Vernetzungspotenzial freigesetzt und Nutzerbedürfnisse aktiv angesprochen werden. Positionen und Bedürfnisse anderer Gesundheitsstakeholder sollten einbezogen werden. Zudem ist sicherzustellen, dass ein nationales Gesundheitsportal jene Sichtbarkeit und Akzeptanz unter Bürger*innen erhält, die für seine Aufgabe unerlässlich sind.
